# Influence of Culture Period on Osteoblast Differentiation of Tissue-Engineered Bone Constructed by Apatite-Fiber Scaffolds Using Radial-Flow Bioreactor

**DOI:** 10.3390/ijms222313080

**Published:** 2021-12-03

**Authors:** Kitaru Suzuki, Jun Fukasawa, Maiko Miura, Poon Nian Lim, Michiyo Honda, Tomokazu Matsuura, Mamoru Aizawa

**Affiliations:** 1 Department of Applied Chemistry, School of Science and Technology, Meiji University, 1-1-1 Higashimita, Tama-ku, Kawasaki 214-8571, Kanagawa, Japan; kitaru_suzuki@meiji.ac.jp (K.S.); jun.fukasawa1985@gmail.com (J.F.); m.miura0107@gmail.com (M.M.); michiyoh@meiji.ac.jp (M.H.); 2 International Institute for Materials with Life Functions, Meiji University, 1-1-1 Higashimita, Tama-ku, Kawasaki 214-8571, Kanagawa, Japan; poonnian@gmail.com (P.N.L.); matsuurat@jikei.ac.jp (T.M.); 3 Department of Laboratory Medicine, The Jikei University School of Medicine, 3-25-8 Nishi-Shinbashi, Minato-ku, Tokyo 105-8461, Japan

**Keywords:** hydroxyapatite, calcium phosphate, apatite-fiber scaffold, rat bone marrow stem cells, tissue-engineered bone, bioreactor

## Abstract

With the limitation of autografts, the development of alternative treatments for bone diseases to alleviate autograft-related complications is highly demanded. In this study, a tissue-engineered bone was formed by culturing rat bone marrow cells (RBMCs) onto porous apatite-fiber scaffolds (AFSs) with three-dimensional (3D) interconnected pores using a radial-flow bioreactor (RFB). Using the optimized flow rate, the effect of different culturing periods on the development of tissue-engineered bone was investigated. The 3D cell culture using RFB was performed for 0, 1 or 2 weeks in a standard medium followed by 0, 1 or 2 weeks in a differentiation medium. Osteoblast differentiation in the tissue-engineered bone was examined by alkaline phosphatase (ALP) and osteocalcin (OC) assays. Furthermore, the tissue-engineered bone was histologically examined by hematoxylin and eosin and alizarin red S stains. We found that the ALP activity and OC content of calcified cells tended to increase with the culture period, and the differentiation of tissue-engineered bone could be controlled by varying the culture period. In addition, the employment of RFB and AFSs provided a favorable 3D environment for cell growth and differentiation. Overall, these results provide valuable insights into the design of tissue-engineered bone for clinical applications.

## 1. Introduction

In orthopedic surgery, autologous bone graft is the gold standard treatment for repairing bone tissue damage [1]. This approach involves osteoinduction, which is important for bone defect treatment [2]. Although autografts are the preferred technique in clinical sites, two major autografting-associated problems remain to be addressed: (i) Limited amount of the grafted bone and (ii) secondary invasion of the healthy bone tissue [1]. Allografts can solve both of these problems. However, they may lead to graft rejection or infection [3]. These limitations lead to a strong impetus for developing alternative treatments for bone regeneration.

Hydroxyapatite (Ca_10_(PO_4_)_6_(OH)_2_; HAp) is a promising artificial bone material due to its biocompatibility and osteoconductivity [2,4,5]. In addition, tissue engineering has been successfully applied to bone regeneration in recent years and could even repair tissues that are difficult to treat with materials alone [6]. In tissue engineering techniques, a combination of three factors: Scaffold, the cells, and growth factors, is important for the reconstruction of the target tissue.

Living human tissues possess complicated three-dimensional (3D) structures [7]. When the bone defect is large, it is difficult to treat using artificial bone materials alone. In static culture, since cells are mainly present on the surface, achieving a uniform cell distribution on the 3D scaffold is difficult [8,9]. In addition, static culture causes difficulty in nutrient exchange, which adversely affects the growth and differentiation of cells [10]. Therefore, seeding cells evenly on the scaffold and differentiating them are critical prior to their implementation to the bone defect area [11]. Using a tissue engineering approach, we aimed to construct a large 3D tissue-engineered bone using a bioreactor.

Bioreactors are used not only in tissue engineering but also in various fields, such as pharmaceuticals [12]. They have been used in the reconstruction of artificial liver, trachea (soft tissue), and bones [13,14,15]. In addition, they can support the circulation of nutrients and excretion of waste products. Therefore, they can be used to create a favorable environment for cell growth. Combining bioreactors and 3D scaffolds make culture conditions suitable for cell growth, thereby helping in reproducing the original 3D structure of living tissues.

Saito et al. [16] reported liver tissue reconstruction using a bioreactor. The organ reconstruction requires the maintenance of viable cells at a high density and co-culture under conditions favorable to several different cell types involved. Therefore, a bioreactor that promotes 3D growth in a high-density perfusion culture has been proposed for the reconstruction of a liver organoid. They have successfully co-cultured three cell types using a radial-flow bioreactor (RFB). In addition, the co-cultured cells exhibited the ability to produce urea. These organ reconstruction technologies contribute greatly to the development of artificial organ transplantation.

Previously, we have successfully synthesized single-crystal apatite fibers (AFs) for the promotion of bone tissue reconstruction [17]. The AFs have a structure similar to the biological bone with a characteristic shape, which exposes much of the Miller index of (300) corresponding to the *a*-plane [18], promoting acidic protein absorption by differential surface charge [19]. Using the AFs, we have developed porous apatite-fiber scaffolds (AFSs) for bone tissue engineering. The AFSs have 3D interconnected pores, involving macropores and micropores. Macropores provide sufficient space for cell growth and proliferation, and micropores facilitate cell differentiation via cell–cell networks [20]. Furthermore, we have constructed a 3D tissue-engineered bone by culturing AFSs with rat bone marrow stem cells (RBMCs) using a RFB. It was reputed that the medium flow rate could affect osteoblast differentiation in a dynamic environment using a RFB [21,22]. Therefore, the AFSs could be suitable for repairing bone defects.

In recent years, it was reported that the mechanical stimuli generated from bioreactors affected bone differentiation [23]. Although we have already optimized the medium flow rate of RFB [21,22], the optimal “period” for cell culture remains unclear. Therefore, we assumed that the culture period was associated with mechanical stimulation. In this study, we examined the effect of the culture period on the osteoblast differentiation of the 3D tissue-engineered bone. Under the optimized flow rate, we evaluated the differentiation of cells seeded onto AFSs at various culture periods.

## 2. Results

### 2.1. AFS Properties

**Figure 1:**
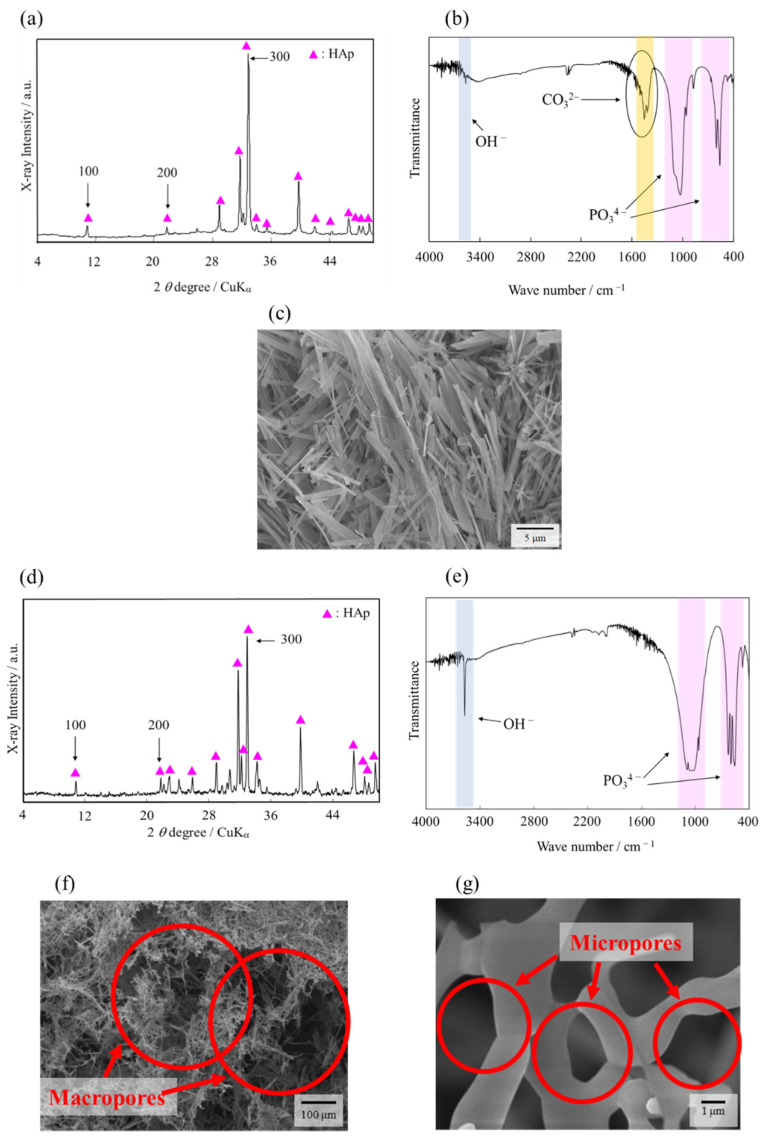
Characterization of AFs and AFS2000. (**a**) XRD pattern, (**b**) FT-IR spectra, (**c**) morphology of AFs, (**d**) XRD pattern, (**e**) FT-IR spectra, and (**f**) microstructure of AFS2000 at low and (**g**) high magnifications. The vertical axis in (**a**,**d**) is shown as an arbitrary unit (a.u.).

X-ray diffraction (XRD) patterns and spectra of the Fourier transforms infrared spectrometry (FT-IR) of AFs are shown in Figure 1a,b. The XRD patterns indicated that AFs were single-phase HAp (Figure 1a). The XRD patterns of AFs showed a strong 300 reflection peak compared to those of HAp (HAp listed in ICDD card #9-432). Figure 1b shows the FT-IR spectra of AFs. The result revealed that AFs had a characteristic functional group of HAp structure. The observed peaks were attributed to the PO_4_^3−^ group and OH^−^ group. Furthermore, it showed the CO_3_^2−^ group due to the undergoing hydrolysis of urea by the homogeneous method. This result indicates that the synthesized AFs were CO_3_ HAp. The type of content seems to be type AB of CO_3_ HAp based on the previous reports [18,24]. The OH^-^ groups in AFS (AFS2000) are sharper than those in AFs. This result may be due to the desorption of carbonate groups and subsequent introduction of the OH^-^ groups by the firing of the AFS2000 in a steam stream. The morphology of AFs was observed by the scanning electron microscopy (SEM) (Figure 1c). From the XRD patterns and SEM images, we can see that the AFs consisted of fiber-shaped particles as in the Figure 1c with a preferred orientation to (300), corresponding to the *a*-plane of HAp.

Figure 1d shows that the XRD patterns of AFS2000 remained as a single-phase HAp, still after firing at 1300 °C for 5 h. In addition, the FT-IR spectra of AFS comprised of the characteristic functional groups of the HAp structure (Figure 1e) without the CO_3_^2−^ peak. This might due to the desorption of CO_3_^2−^ from apatite crystals during sintering. Moreover, the OH^−^ peak had a sharp peak relative to the AFs spectra. The steam during firing could have replaced the CO_3_^2−^ group, which disappeared during firing, with the OH^−^ peak. The SEM images of AFS2000 shown in Figure 1f,g confirmed that the scaffolds had macropores, which were formed due to the burning out of carbon beads with a diameter of ~150 μm, while micropores were formed by the intertwining individual AFs.

### 2.2. Cell Viability

**Figure 2:**
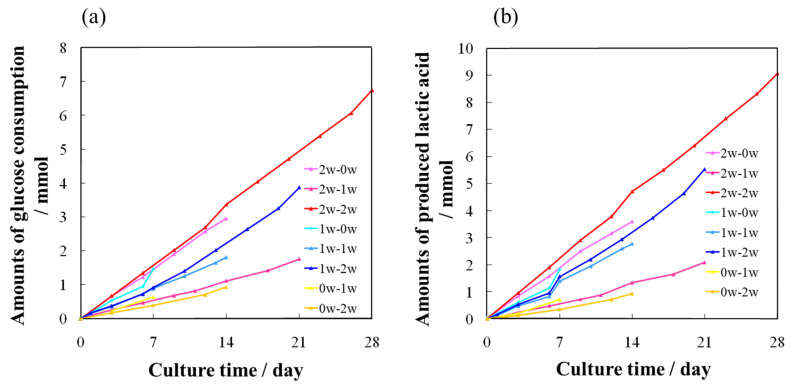
Culture conditions during the 3D culture using a RFB. (**a**) Glucose consumption and (**b**) lactic acid production with the culture period.

When the 3D culture was performed using the RFB with AFS2000, the amounts of glucose consumption and lactate production in the culture medium were measured. In general, cells consume glucose and produce lactic acid. The results are shown in Figure 2a,b. The amount of glucose consumption increased in both the standard and differentiation medium. The amount of lactate production increased in response to the consumption of glucose. These results indicated that RBMCs metabolized glucose, and the favorable 3D culture environment provided by RFB with AFSs promoted the cell proliferation of RBMCs.

### 2.3. Histological Evaluations from AFSs after 3D Cell Culture of RBMCs Using a RFB

To examine cellular localization, frozen sections were prepared from AFSs after 3D cell culture using a RFB, and were histologically examined with hematoxylin and eosin (HE) staining. Table 1 presents the culture periods using the standard and differentiation medium.

**Figure 3:**
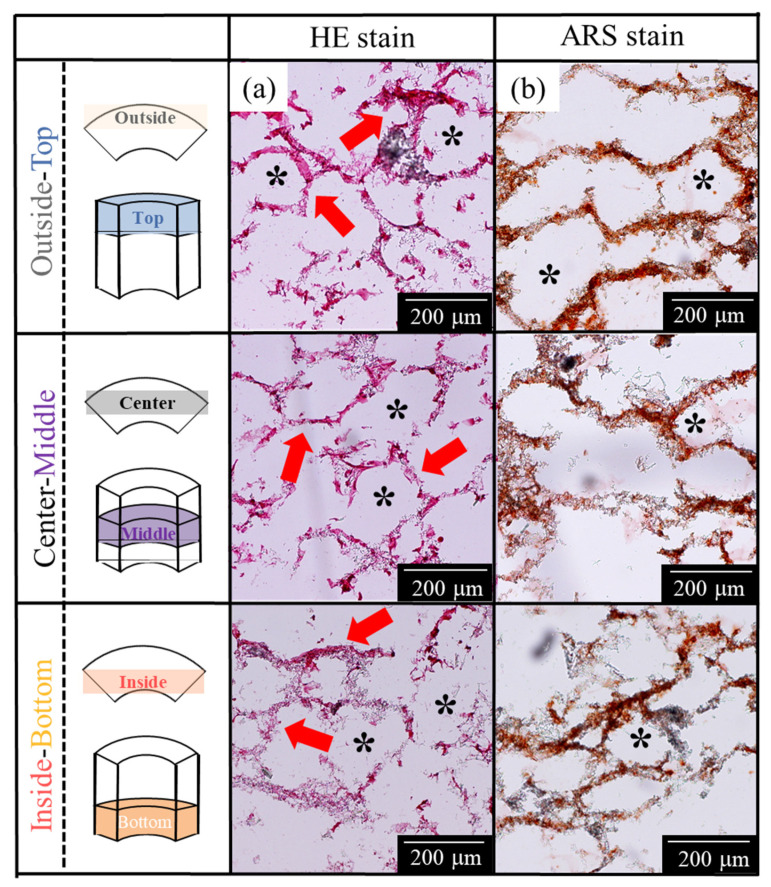
Histological evaluation using HE and ARS staining for the “1w-1w”. (**a**) HE staining and (**b**) ARS staining. Red arrows: Cells located along the AFS skeletons; *: AFS macropores.

The results of the “1w-1w” are shown in Figure 3a,b. The results of HE staining shown in Figure 3a indicated that the cells in AFSs were present along the AFS skeleton. In addition, the cell penetration into the inside of the AFSs could be observed. The cells were located along the pores formed by the burning of carbon beads. Moreover, the cells tended to be more localized outside of the AFSs. However, the cells were almost distributed in similar sites at all areas of AFSs (Figure 3a). The results of alizarin red S (ARS) staining sections are shown in Figure 3b. As shown in Figure 3b, calcified cells were observed in all the sections of AFSs.

**Figure 4:**
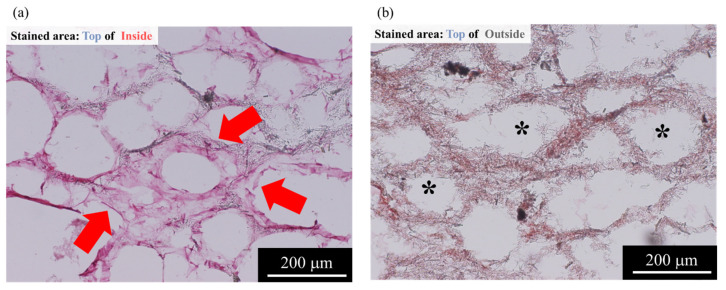
High-magnification histological evaluation using HE and ARS staining for “2w-2w”. (**a**) HE staining and (**b**) ARS staining. Red arrows: Cells located along the AFS skeletons; *: AFS macropores.

Furthermore, the results that performed the HE staining of the “2w-2w” are shown in Figure 4a. The “2w-2w”, which was the longest period sample in this study, showed that cells were penetrated into the macropores. In addition, more calcified cells were observed in the “2w-2w” (Figure 4b). Therefore, macropores produced from the burning of carbon beads in AFSs may provide a good microenvironment for cells to attach and promote cell growth.

### 2.4. Quantitative Evaluations of Bone Differentiation Markers for AFS-Cultured RBMCs Using a RFB

**Figure 5:**
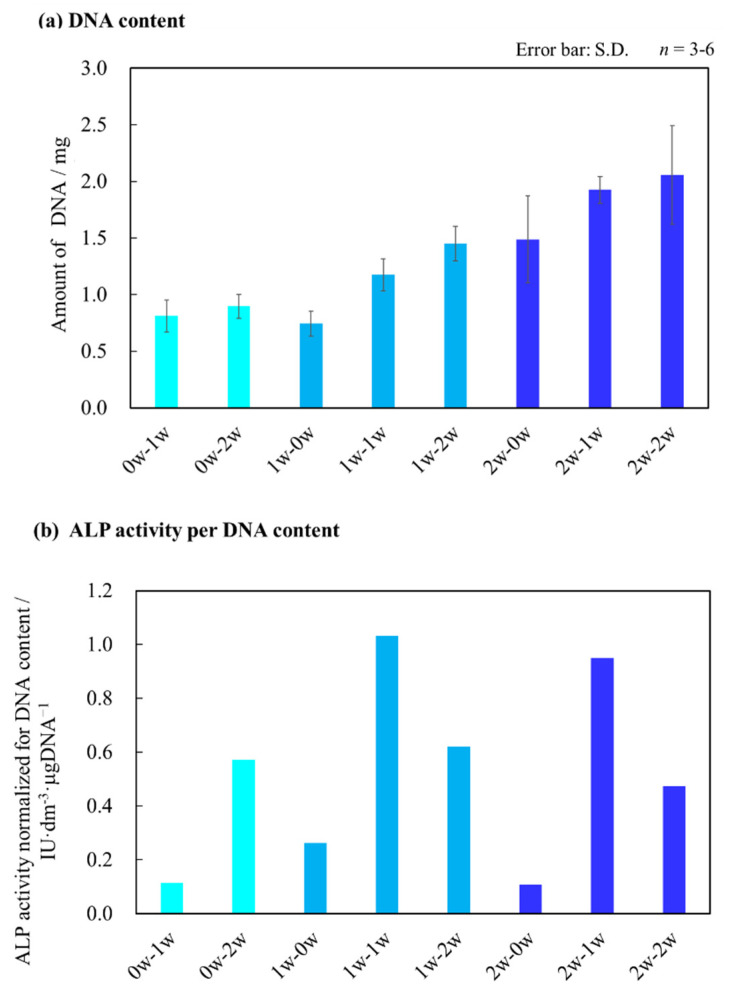

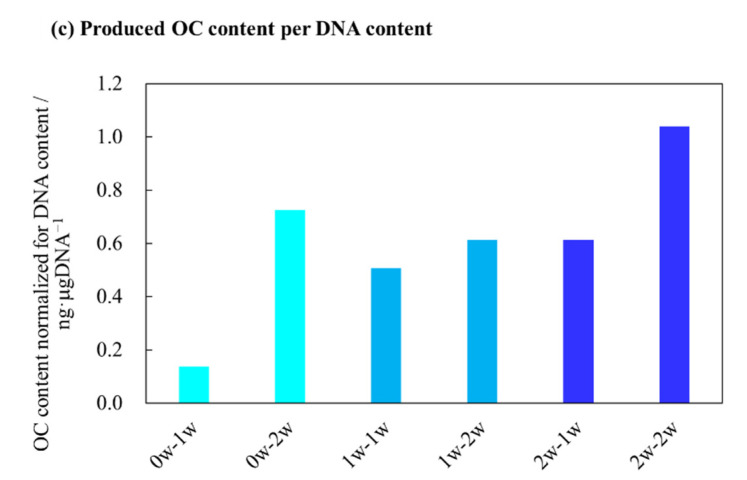
Biological assays of tissue-engineered bones constructed under various cell culture conditions. (**a**) Amount of DNA in 1/4 AFS, (**b**) ALP activity normalized for the amount of DNA *, and (**c**) total production amount of OC normalized for the amount of DNA *. *: The DNA amount, ALP activity value, and OC production amount in 1/4 size AFS were multiplied by 4 and calculated as the result of the whole AFS. Therefore, no error bars were added to the normalized graphs. The error bars in (**a**) are shown as standard deviation (S.D.).

Bone differentiation markers, such as alkaline phosphatase (ALP) and osteocalcin (OC), were normalized for DNA contents in AFSs to reveal the osteoblast differentiation stage in AFSs. As shown in Figure 5a–c, experiments were performed under eight different culture conditions. With the increasing number of culture days, the amount of DNA also tended to increase in all the samples (Figure 5a).

In Figure 5b, in the case of the incubation period of 1 w using the differentiation medium, the results of the ALP activity values normalized by the amount of DNA indicated that the ALP activity values in the “1 w series” tended to be higher than the 0 and 2 w series, with the “1w-1w” as the highest ALP activity value.

The results of the quantitative assay of the six samples for the amount of OC are shown in Figure 5c. The results showed that the production amount of OC normalized by the amount of DNA tended to be higher in the samples using the differentiation medium for a relatively long period, and the series with the longest culture period (2w-2w) showed the highest values (Figure 5c). In addition, when comparing the OC produced under the same differentiation culture period, such as “1w-2w” and “2w-2w”, the production amount of OC tended to increase in the series with a longer incubation period using the standard medium.

## 3. Discussion

We have previously employed the combination of AFS and RFB, which successfully developed a tissue-engineered bone. In this study, we used both the standard medium to grow RBMCs and the differentiation medium to differentiate RBMCs, and focused on the period of each culture.

First, we evaluated the cell viability of tissue-engineered bone. In general, when the cells expand in the scaffold, cells will consume glucose to produce lactic acid.

Our results in Figure 2 indicated that the RBMCs metabolized glucose due the fact that when the cells expand in the scaffold, they consume glucose to produce lactic acid [25], confirming that the cells were successfully 3D cultured in AFSs using a RFB. Moreover, this phenomenon has been maintained over a period of 4 weeks. Therefore, the favorable culture environment provided by the RFB with AFSs promoted the cell proliferation of RBMCs.

The tissue-engineered bone, “2w-2w”, had the largest production of DNA (Figure 5a). When the culture medium was changed to the differentiation medium to culture for a long period, the amount of DNA also tended to increase. However, the increment of the DNA amount for “0w-2w” cultured using differentiation medium conditions tended to be lower than “2w-0w”, which was cultured in the standard medium. These differences might be due to the insufficient cell expansion before inducing the mesenchymal stem cells. From the results of cell viability and quantitative evaluation of DNA amount, this culture system could achieve the culturing of 3D-structure scaffold for 4 weeks, despite the fact that it was performed under in vitro conditions.

In the qualitative evaluation for a frozen section by HE staining, we could observe the cell penetration. The cells penetrated into the pores, which were created by the burnt carbon beads. Although the scaffolding material used in this study had a height of 15 mm, a relatively uniform cell distribution was achieved in the tissue-engineered bone (Figure 4a). Furthermore, for the cells that were cultured up to 4 weeks, good cell growth was observed. Therefore, the combination of AFS with high porosity using a RFB may enhance the circulation of the medium and nutrients.

Then, bone differentiation properties for the tissue-engineered bones were assayed. The quantitative evaluation of bone differentiation marker revealed bone differentiation properties in the tissue-engineered bone. In Figure 5b, the ALP activity in the “1 w series” was higher than those in the “0 w series” and “2 w series”, with the “1w-1w” as the highest. Furthermore, the tissue-engineered bone cultured using the differentiation medium for only 1 week had the highest ALP activity. These results implied that we constructed a tissue-engineered bone, which demonstrated an early stage of bone differentiation. On the other hand, the ALP activity of the “1w-2w” decreased. These results suggested that the mesenchymal stem cells might induce a late-stage bone differentiation.

Figure 5c shows the production amount of OC with a longer culturing period, in which the tissue-engineered bone would increase the production of OC. In particular, the “2w-2w” produced more OC than the other samples, as shown in Figure 5c, with the “2w-2w” as the greatest OC production. In addition, when comparing the OC produced under the same differentiation culture period, such as “1w-2w” and “2w-2w”, the production amount of OC tended to increase in the series with a longer incubation period using the standard medium. Based on the results of the production of DNA, these differences might be due to the cell number in the tissue-engineered bone. These results illustrated the influence of the environment on cells in the scaffolding material, where bone differentiation could be promoted. Therefore, we could also construct the tissue-engineered bone with late-stage bone differentiation.

Previous experiments [21,22] by our group have shown that the differentiation of mesenchymal stem cells in the scaffold material can be promoted by applying an appropriate mechanical shear stress, such as the optimized flow rate of the cell culture medium. In our RFB system using AFS, it was 6.3 cm^3^ min^−1^. The detailed results have been reported in other studies [21,22]. In addition, Holtorf et al. [26] reported the effect of flow perfusion culture on bone differentiation using titanium fiber mesh scaffolds. They reported that the bioreactor had a two-fold contribution to cell differentiation: (i) Promoting mass transport to the scaffold interior and (ii) mechanically stimulating cells due to the fluid shear force within the scaffold. Janssen et al. [27] reported that the shear stress generated by the bioreactor helped in the production of the extracellular matrix. Furthermore, they also reported that the cultivation of bone marrow stem cells on 3D constructs in perfusion bioreactors enhanced the growth, differentiation, and mineralized matrix production in vitro. Our results were in line with these studies, and indicated that the 3D tissue-engineered bone in the late-stage bone differentiation was achieved using AFSs cultured in the RFB. Furthermore, from the results of ARS staining, calcification cells were also observed throughout the “1w-1w” and “2w-2w”. The long-term culture using AFSs and the RFB enhanced the cell growth and differentiation of mesenchymal stem cells into the late stage of bone lineage. The “1w-1w” may have a high bone-forming potential since the results of the ALP activity were higher relatively, and calcification was observed despite the early culture period. Therefore, the use of a bioreactor promoted the uniform cell attachment on the scaffold and helped in the circulation of nutrients and waste products. The shear stress caused by the flow of medium during the RFB culture may promote osteoblast differentiation. Moreover, the macropores and micropores of AFSs could also promote bone differentiation by providing a favorable environment for osteogenesis [28]. It was reported that a pore of ~100 μm was required for bone growth [29,30], and the size of the interconnection should be at least ~50 μm [31]. This was due to the fact that the micropores promoted the bone formation by providing nutrients and cell–cell communication [32], and increased the contact area between the cells and the bone [33,34]. Taken together, this study demonstrated that culturing mesenchymal stem cells using a RFB is an effective method for treating bone defects as it could maintain a uniform culture environment, even in a large scaffolding material. In summary, we have successfully constructed the 3D tissue-engineered bone with various bone differentiation properties by controlling the culture period using a RFB.

## 4. Materials and Methods

### 4.1. Fabrication and Characterization of AFSs

AFs were synthesized via a homogeneous precipitation method using urea, as described previously [18,24]. AFs were suspended with ~150 μm diameter spherical carbon beads in a mixed solvent (ethanol/water = 1:1 [*v*/*v*]) at an AF/carbon bead [*w*/*w*] ratio of 1/20 [17,21,22]. The carbon beads mixed with the AF slurry were used as a pore-forming agent.

**Figure 6:**
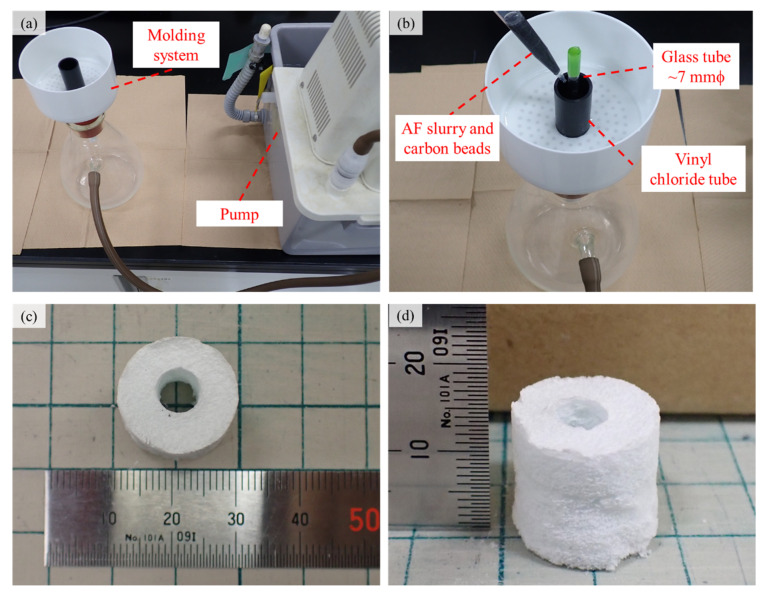
Modeling system for AFS2000. (**a**) Overview and (**b**) detailed view and photographs of AFS2000. (**c**) Top and (**d**) side views.

Figure 6a,b shows the modeling system using the vinyl chloride tube mold and glass tube. The green compacts for scaffolds were fabricated by pouring the carbon bead containing AF slurry into a vinyl chloride tube (~φ20 mm) mold. A glass tube (outer diameter of ~7 mm) was placed at the center of the vinyl chloride tube mold, and the slurry containing AFs and carbon beads (18 cm^3^) was poured with a micropipette and vacuumed by a pump. After the vacuuming process with the pump, the compacts were fired at 1300 °C for 5 h (hearting rate: 5 °C min^−1^) using an electric furnace (KTF433W, KOYO, Nara, Japan) in a steam atmosphere to produce AFSs, referred to as “AFS2000”.

In this study, we used AFS2000 with a diameter of ~φ18 mm and a height of ~15 mm, as shown in Figure 6c,d. The crystalline phase of scaffolds was identified by XRD (Miniflex, Rigaku, Tokyo, Japan) equipped with a CuK_α_ radiation source at 30 kV and 15 mA, and the data were collected under the following conditions: 2*θ* range of 4–50°, scan rate of 4° per minute, and sampling width of 0.04°. The functional groups of AFSs were detected using FT-IR (IR Prestige-21, Shimadzu, Kyoto, Japan) in the range of 400–4000 cm^−1^ with a spectral resolution of 4 cm^−1^. The FT-IR samples were prepared by mixing the sample and KBr powders and compressing them into discs. The microstructure of AFSs was observed by SEM (JSM6390LA, JEOL, Tokyo, Japan) at 10 kV. The SEM samples were prepared by fixing the ceramics on double-sided carbon tapes and depositing platinum particles in a vacuum.

### 4.2. Primary Culture of RBMCs

RBMCs were obtained from the bone marrow of femora and tibiae from 4-week-old male Wistar rats, as previously reported [35]. The bone marrows in the femora and tibiae were flushed out with the α-minimal essential medium (α-MEM) using a syringe. The cells harvested from bone shafts were cultured in a standard medium (α-MEM with 10% fetal bovine serum and antibiotics (100 units cm^−3^ penicillin and 100 μg cm^−3^ streptomycin)) at 37 °C in a humidified atmosphere with 5% CO_2_. The next day, a medium change was carried out to remove the unattached cells. After 8 days, RBMCs were harvested using the trypsin-EDTA solution (Gibco, Thermo Fisher Scientific, Waltham, MA, USA) and a cell scraper (IWAKI, AGC Techno Glass, Shizuoka, Japan). The cells were expanded for 16 days from the harvesting bone marrows, and the cells in passage 3 were used for the experiments. The number of collected cells was 1.0 × 10^7^. The animal treatments were all performed according to the Guidelines for Animal Care and Use Committee of Meiji University (No. MUIACUC 2020-11/Date: 5 June 2020).

### 4.3. The 3D Cell Culture Using a RFB

**Figure 7:**
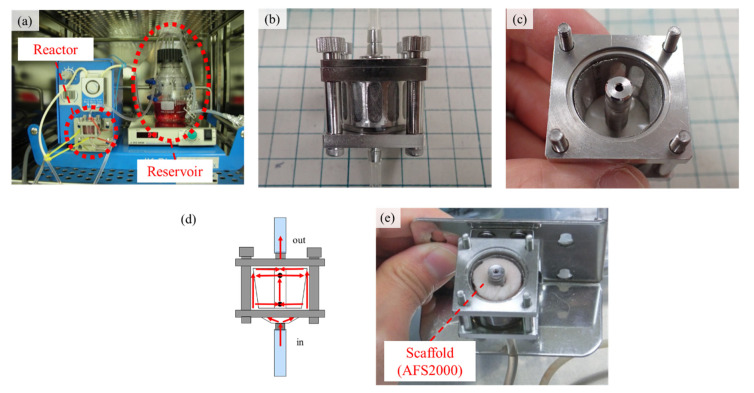
Photographs of a RFB. (**a**) Overview of a RFB, (**b**,**c**) structure of the bioreactor, (**d**) schematic of medium flow in the bioreactor, and (**e**) AFS2000 placed in the RFB.

The AFS2000 was soaked in the standard medium and placed into a RFB on the day before seeding. The RFB system (ABLE, Tokyo, Japan) with a chamber volume of 5 cm^3^ was used for the 3D cell culture of RBMCs (Figure 7). The 1.0 × 10^7^ cells of RBMCs suspended in the 20 cm^3^ medium were injected using a syringe in the reservoir of RFB, then the cell suspension was circulated. The flow rates of the mediums were set to an optimized flow rate at 6.3 cm^3^ min^−1^, and the cells were seeded throughout the AFSs [22]. The 3D cell culture using the RFB was performed in a standard medium for 0, 1, and 2 weeks. After culturing in the standard medium, culturing in a differentiation medium (standard medium containing 10 nmol dm^−3^ dexamethasone, 200 μmol dm^−3^ ascorbic acid, and 1 mmol dm^−3^ β-sodium glycerophosphate) followed for 0, 1, and 2 weeks. We conducted several 3D cell culture periods using the RFB. Table 1 presents the culture periods using the standard and differentiation mediums. The longest culture period was 4 weeks. When the culture was performed using the standard medium for 1 week, it was followed by culturing in a differentiation medium for 2 weeks, denoted as “1w-2w”.

### 4.4. Cell Viability Assay

Referring to the previous report [25], to confirm the proliferation of RBMCs, the levels of glucose and lactic acid in the medium were monitored. The Glu Test Ace R (Arkray, Kyoto, Japan) and Lactate Pro (Arkray, Kyoto, Japan) were used for glucose and lactic acid detection, respectively.

### 4.5. Harvesting the Tissue-Engineered Bone

After cell culture using the RFB, tissue-engineered bones were harvested from the RFB and washed with phosphate-buffered saline (PBS). One-quarter of AFS was cut off for histological evaluation, and three-quarters of AFSs were used for the determination of the DNA amount, ALP assay value, and OC production amount, respectively.

#### 4.5.1. Determination of DNA Amount

**Figure 8:**
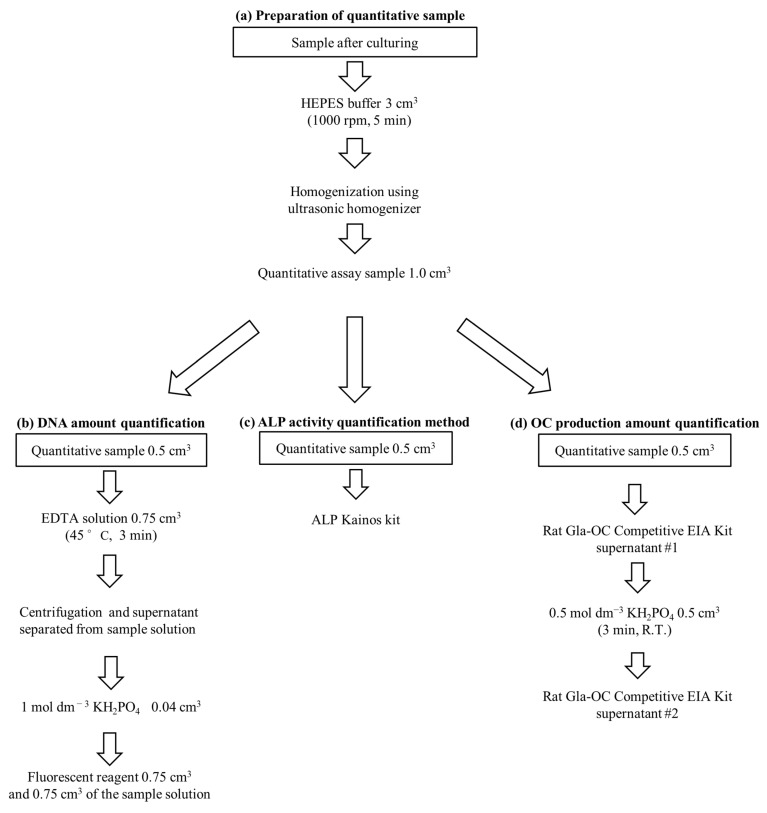
Biochemical evaluations. (**a**) Preparation of quantitative sample for DNA, ALP, and OC assays, (**b**) DNA quantification method, (**c**) ALP activity quantification method *, (**d**) OC production amount quantification method * (the sum of the two values (#1 and #2) were treated as the OC production amount). *: The same quantitative samples (0.5 cm^3^) were used for the ALP and OC assays.

A quarter of tissue-engineered bone was washed with the 3 cm^3^ 4-(2-hydroxyethyl)-1-piperazineethanesulfonic acid (HEPES) solution and was centrifuged three times at 1000 rpm for 5 min. Then, the tissue-engineered bone was stored at −80 °C with the 1 cm^3^ HEPES buffer until the quantitative DNA evaluation was performed. After thawing, the tissue-engineered bone was homogenized using an ultrasonic homogenizer. The solutions were aliquoted to 0.5 cm^3^ each. In addition, the 0.75 cm^3^ 10 mmol dm^−3^ EDTA solution (pH 12.3; adjusted with NaOH) was added to 0.5 cm^3^ of the sample solution and incubated at 45 °C for 3 min. Subsequently, the solutions were centrifuged at 10,000 rpm for 3 min, and the 0.04 cm^3^ 1 mol dm^−3^ KH_2_PO_4_ solution was added to 1 cm^3^ of the supernatant. The DNA amount was measured by mixing 0.75 cm^3^ of the fluorescent reagent solution and 0.75 cm^3^ of the sample solution. In this study, the used fluorescent reagent was prepared by adding 0.01 mmol dm^−3^ Tris (hydroxymethyl) aminomethane and 800 pg cm^−3^ Hoechst 33258 fluorescent reagent to 0.1 mmol dm^−3^ NaCl (Figure 8a,b). The excitation and emission wavelengths were 360 and 460 nm, respectively, and the fluorescence intensity of Hoechst 33258 was measured. The total amount of DNA in AFS was calculated by multiplying the determined value by 4.

#### 4.5.2. Determination of the ALP Activity and OC Amount

The level of bone differentiation in eight types of samples was examined by determining the content of two types of differentiation makers. ALP is the initial/middle stage, and OC is the late stage in bone differentiation toward the osteogenic lineage [36]. We performed the quantitative osteocalcin assay of only six sample types since the tissue-engineered bones that were not cultured using the differentiation medium were excluded from the OC assay. The determination of the ALP activity and OC production amount was performed for the remaining 0.5 cm^3^ sample solution. To examine the ALP activity, we used a measuring kit (ALP Kainos, Kainos, Tokyo, Japan) by the absorption of 500 nm (Figure 8c). The production amount of OC was measured by the Rat Gla-OC Competitive EIA Kit (TaKaRa Bio, Shiga, Japan).

Moreover, we measured the amount of OC contained in the residue by adding 0.5 cm^3^ of 0.5 mol dm^−3^ KH_2_PO_4_ solution. After 3 min, the supernatant obtained through centrifugation was also measured to determine the amount of OC absorbed in the AFSs. The sum of the two values was treated as the production amount of OC in this study (Figure 8d).

### 4.6. Histological Evaluations

The tissue-engineered bones were fixed in 4% paraformaldehyde in PBS for 60 min at 4 °C. Next, the samples were immersed in the 0.1% gelatin solution for 60 min at room temperature and kept in the same solution for 60 min under reduced pressure. Finally, the samples were embedded in an Optimal Cutting Temperature (OCT) compound (SAKURA Tissue-Tek, Tokyo, Japan) and frozen at −20 °C overnight. The frozen specimens were then transferred to −80 °C until section preparation. In addition, the frozen sections with a thickness of 18 μm were cut using a microtome (CM3050 S, Leica, Wetzlar, Germany).

**Figure 9:**
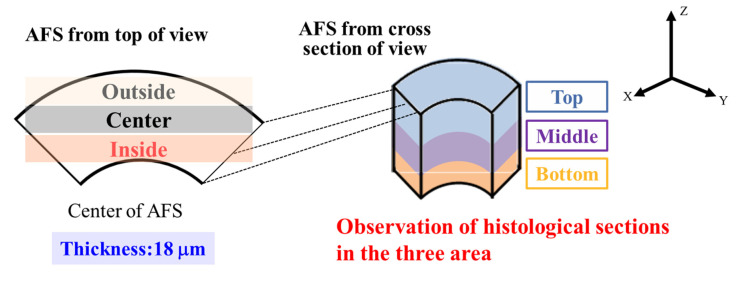
Observation direction of tissue-engineered bone.

HE staining was performed to examine cellular localization. The specimens were soaked in HE solutions. ARS staining was used to confirm the calcification levels in tissue-engineered bone. ARS (FUJIFILM Wako Pure Chemical Industry, Osaka, Japan) was dissolved in pure water and adjusted to pH 6.3 using ammonia water. The specimens were soaked in the ARS solution and washed with ethanol and xylene. The sections, after the previously mentioned staining, were observed using an optical microscope (BX51, Olympus, Tokyo, Japan). For histological evaluation, AFS was divided into top, middle, and bottom in the z-axis direction, as well as divided into outside, center, and inside in x- and y-axis directions for observation (Figure 9).

## 5. Conclusions

To conclude, we have successfully constructed a 3D tissue-engineered bone of large size (~18 mm diameter × 15 mm height) using AFSs cultured in a RFB for potential clinical applications. Furthermore, we showed that bone differentiation could be controlled by changing the culture period. In particular, the “1w-1w” may have high bone-forming potential since the results of the ALP activity were higher relatively, and calcification was confirmed despite the early culture period. These findings are important for bone regeneration and bone disease treatment using tissue engineering approaches.

## Figures and Tables

**Table 1:** Preparation conditions for tissue-engineered bone.

0 w Series	1 w Series	2 w Series
-	1w-0w	2w-0w
0w-1w	1w-1w	2w-1w
0w-2w	1w-2w	2w-2w

For 0 w series, differentiation medium was used directly through culturing process. For 1 w and 2 w series, standard medium was firstly used for 1 or 2 weeks, followed by 0, 1, or 2 weeks with differentiation medium.

## Data Availability

The data that support the findings of this study are available from the corresponding author (M.A.) upon reasonable request.
